# Ultra‐Broad Emission Copper Halide Scintillator‐Based X‐Ray Imager

**DOI:** 10.1002/advs.202405995

**Published:** 2024-11-26

**Authors:** Haocheng Lv, Wenyi Shao, Haifeng Chen, Guoyang Zhu, Yao Wang, Zhenzhong Zhang, Hongwei Liang

**Affiliations:** ^1^ Dalian Key Laboratory of Wide Band Gap Semiconductor Devices Integration and System, School of Integrated Circuits Dalian University of Technology Dalian 116024 China

**Keywords:** emerging copper halide, scintillator, ultra‐broad emission, X‐ray imager

## Abstract

Lead‐free metal‐halide scintillators are gaining considerable attention as more eco‐friendly and superior alternatives to their lead‐based counterparts. However, novel broad‐emission band scintillators like the state‐of‐the‐art CsI: Tl scintillator, which can generate high signals due to its strong compatibility with the spectral responsivity of regular photodiode arrays, are still less investigated. Herein, a TPA_2_Cu_2_I_4_ (TPACI) copper halide scintillator with a unique ultra‐broad emission (FWHM > 240 nm) is developed, which shows universal compatibility with the peak response range of commercial photodetector. The optical properties characterization and mechanism analysis indicates that this ultra‐broad spectrum can be attributed to the dual self‐trapped exciton (STE) emission consisting of two emission bands. Benefiting from the large Stokes shift and ultra‐broad emission band enabled by the dual STE, the self‐absorption‐free TPACI scintillator exhibits efficient white light emission with a high photoluminescence quantum yields of 94.27%, a high light yield of ≈40124 photons MeV^−1^. Moreover, a prototype of a TPACI scintillator‐based X‐ray imager is assembled for inspecting the internal structures of biological and electronic devices, which demonstrated a high resolution of 5.5 lp mm^−1^ at modulation transfer function = 0.2. These findings provide insights into the design of efficient, broad‐emission scintillators for high‐resolution X‐ray imaging.

## Graphical abstract

A novel copper halide scintillator with a unique ultra‐broad emission band demonstrates universal compatibility with the peak response range of commercial photodetector. A prototype X‐ray imager based on this copper halide scintillator is constructed for inspecting the internal structures of biological and electronic devices with a high resolution of 5.5 lp mm^−1^ at modulation transfer function = 0.2.

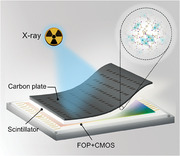

## 1 Introduction

The success of indirect radiation detection strategies relies on the development of high‐performance scintillator materials.^[^ ^1^, ^2^ ^]^ As the heart of the detector, the scintillator enables the conversion of incident radiation/particles into ultraviolet or visible light, which can be collected by the photodetector and read out by the electronics system to generate an image, reflect energy information, and measure the radiation dose.^[^ ^3^, ^4^, ^5^ ^]^ Current commercial photodetectors, including charge‐coupled device (CCD), complementary metal−oxide−semiconductor (CMOS), photomultiplier (PMT), and silicon photodiode (SiPD), exhibit different quantum efficiencies (QE) over a range of spectral responses. The selection of photodetector type depends on the application scenario, e.g., CMOS for flat panel detectors, SiPD for line array detectors, and PMT for energy detection. In this case, the apparent light output (ALO) of the scintillator with specific emission spectra when integrated with different types of photodetectors can be briefly expressed as^[^ ^6^ ^]^:

(1) $$\;ALO=\int I_{i}\left(\lambda \right)S\left(\lambda \right)d\lambda$$

where *I_i_* (λ) is the radioluminescence (RL) intensity of scintillators, and *S*(λ) is the wavelength‐dependent QE of the given photodetector. This suggests that broad‐emission scintillators can provide better universal compatibility to the spectral response of different photodetectors when scintillators with different emission spectra are normalized. This well‐compatibility with the spectral responsivity of photodetectors enables the efficient collection of scintillation photons. Therefore, the development of broad‐emission scintillators is urgently needed, but this category of scintillators is rarely reported.

The 3d^10^ electronic configuration of copper halide can yield a variety of coordination numbers/geometries and multiple connection modes with halogens, resulting in great structural diversity and a variety of photophysical properties for the copper iodide family. Their potential applications as phosphor‐converted WLEDs,^[^ ^7^, ^8^ ^]^ electroluminescent LEDs,^[^ ^7^, ^9^, ^10^ ^]^ anti‐counterfeiting,^[^ ^11^, ^12^ ^]^ and X‐ray scintillators^[^ ^13^, ^14^, ^15^, ^16^ ^]^ have been recently reported. Copper halide systems are emerging candidates for X‐ray scintillators due to their high atomic number, no self‐absorption, high photoluminescence quantum yields (PLQY), and absence of toxic elements. Among them, Cs_3_Cu_2_I_5_ has been extensively studied as an exemplary scintillator due to its excellent scintillation performance.^[^ ^17^ ^]^ However, the emission spectrum of Cs_3_Cu_2_I_5_ in the blue light band seems to match only the peak QE based on PMT in radiation or particle energy detection. Instead, it exhibits a large mismatch for the peak QE of typical silicon‐based photodetectors employed in X‐ray imaging, leading to a low scintillation photon collection efficiency. Although the spectral emission range can be manipulated through doping, structural, and component modulation strategies,^[^ ^18^, ^19^ ^]^ such modifications are usually limited while potentially degrading the intrinsic light output and material stability.

Herein, we developed a TPA_2_Cu_2_I_4_ (TPACI) copper halide scintillator with a unique ultra‐broad emission. The full width at half‐maximum (FWHM) of its emission band can exceed 240 nm, almost overlapping the visible region, which shows universal compatibility with the peak response range of commercial photodetectors. TPACI emits a bright white light consisting of dual emission bands with a PLQY of 94.27% at room temperature, deriving from the radiative recombination of the dual self‐trapped exciton (STE) in [Cu_2_I_4_]^2−^ clusters. Extending to the X‐ray scintillator application, TPACI exhibits a high light yield of 40124 photons MeV^−1^, a low detection limit of 126 nGy_air_ s^−1^, and a low afterglow level. In addition, it shows scintillation light output stability under a 6 MeV hard X‐ray irradiation with a total dose rate of 10 Gy from a medical radiotherapy accelerator. As a conceptual demonstration, a prototype of a TPACI scintillator‐based X‐ray imager was assembled for inspecting the internal structures of biological and electronic devices, yielding a resolution of 5.5 lp mm^−1^ at modulation transfer function (MTF) = 0.2.

## 2 Results and Discussion

**Figure 1:**
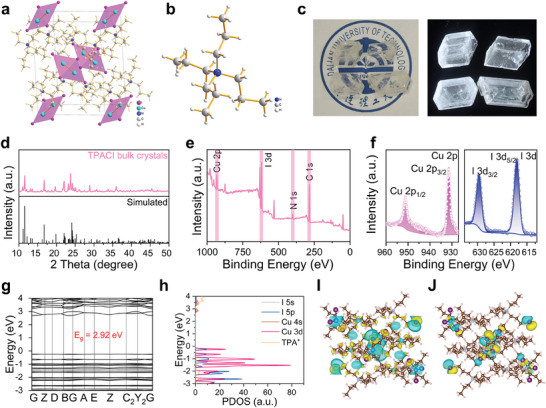
Material characterization of the TPACI bulk crystals. a) Diagram of TPACI crystal structure and organic cation of TPA^+^ b). c) Photograph of the TPACI bulk crystals under daylight and 365 nm UV excitation. d) Experimental PXRD and simulated SCXRD results of the TPACI bulk crystals. e) XPS spectra of the TPACI bulk crystals. f) High‐resolution X‐ray photoelectron spectroscopy (XPS) of Cu 2*p* and I 3*d* in the TPACI bulk crystals. g) Band structure and atoms projected density of states (PDOS) h) of TPACI. i,j) electronic charge density for the conduction band minimum (CBM) and valence band maximum (VBM).

To comprehend the crystal structure of this novel material, TPACI bulk crystals were synthesized through a straightforward solution processing method. The crystal structure of TPACI was determined using SCXRD. In **Figure** **1**a, the crystal system of TPACI is depicted within the monoclinic space group P21/n. The crystallographic information file (CIF) and the crystal structure parameters of TPACI can be found in Table S1 (Supporting Information). The characteristic [Cu_2_I_4_]^2−^ clusters are spatially separated and encircled by monovalent TPA^+^ cations, forming a periodic 0D structure. In addition, the neighboring copper atoms are bonded to four iodine atoms within a plane, and two copper atoms occupy a single site, creating an ideal parallelogram geometry. The closest Cu‐Cu distance within a [Cu_2_I_4_]^2−^ cluster is 2.62 Å, which is shorter than the 2.72 Å observed in (TBA)_2_Cu_2_I_4_ and the 3.12 Å in (MA)_4_Cu_2_Br_6_, indicating a robust Cu–Cu interaction in TPACI SCs.^[^ ^20^, ^21^ ^]^ The structure of TPA^+^ is shown in Figure 1b. Figure 1c exhibits the optical photographs of TPACI bulk crystals. Via a solution slow evaporation method, TPACI bulk crystals are uniform and have a diameter of over 1 cm, which is transparent in the ambient atmosphere, suggesting practically no absorption in visible light. Under UV excitation, TPACI bulk crystals show bright‐white emission. The PXRD pattern of bulk TPACI crystals aligns with the simulated results derived from SCXRD data (Figure 1d), affirming the crystal structure and high phase purity of the prepared sample. The XPS is applied to identify elemental species of the TPACI bulk crystals (Figure 1e). Figure 1f shows the high‐resolution XPS results of Cu 2*p*, I 3*d* in the TPACI bulk crystal. The Cu 2*p* XPS results show the binding energies of Cu 2*p*_3/2_ and Cu 2*p*_1/2_ located at 931.5 and 951.2 eV, respectively, consistent with the monovalent Cu states instead of the divalent Cu^2+^ states (942.4 and 962.3 eV). It indicates that oxidation from Cu^+^ to Cu^2+^ states does not occur during the growth process of bulk crystals, demonstrating the chemical stability of the obtained bulk crystals. The FTIR findings depicted in Figure S1 (Supporting Information) illustrate the asymmetric C─H stretching and symmetric C─H stretching of the −CH_3_ group in the Organic cation of TPA^+^.^[^ ^22^ ^]^ To get better insight into the electronic properties of the TPACI, their electronic structure, PDOS, and partial charge density maps were conducted. The Density functional theory (DFT) calculations show that TPACI has a direct bandgap characteristic with a value of 2.92 eV (Figure 1g). The valence band is nearly flat and discrete, due to the separation of the [Cu_2_I_4_]^2−^ clusters by the large organic molecules.^[^ ^23^ ^]^ The valence and conduction bands of the TPACI indicate the strong quantum confinement effect, and it's also reflected in the (C_16_H_36_N)_2_Cu_2_I_4_, demonstrating a typical feature of 0D metal halides.^[^ ^24^ ^]^ The conduction band exhibits greater dispersion compared to the valence band. Consequently, the holes are localized at the [Cu_2_I_4_]^2−^ clusters, whereas electrons display a higher degree of delocalization, which delays electron–hole recombination, leading to a prolonged exciton lifetime.^[^ ^23^ ^]^ Figure 1h illustrates the PDOS of TPACI. The computed DOS shows that the valence band is mainly dominated by Cu‐d and I‐p states and the conduction band is mainly composed of Cu‐s and I‐p orbitals. Hence, it is anticipated that excitons tend to localize on the isolated [Cu_2_I_4_]^2−^ clusters. The charge density distributions associated with the valence band maximum (VBM) and the conduction band minimum (CBM) further indicate that the electron density predominantly stems from the [Cu_2_I_4_]^2−^ clusters (Figure 1i,j).

**Figure 2:**
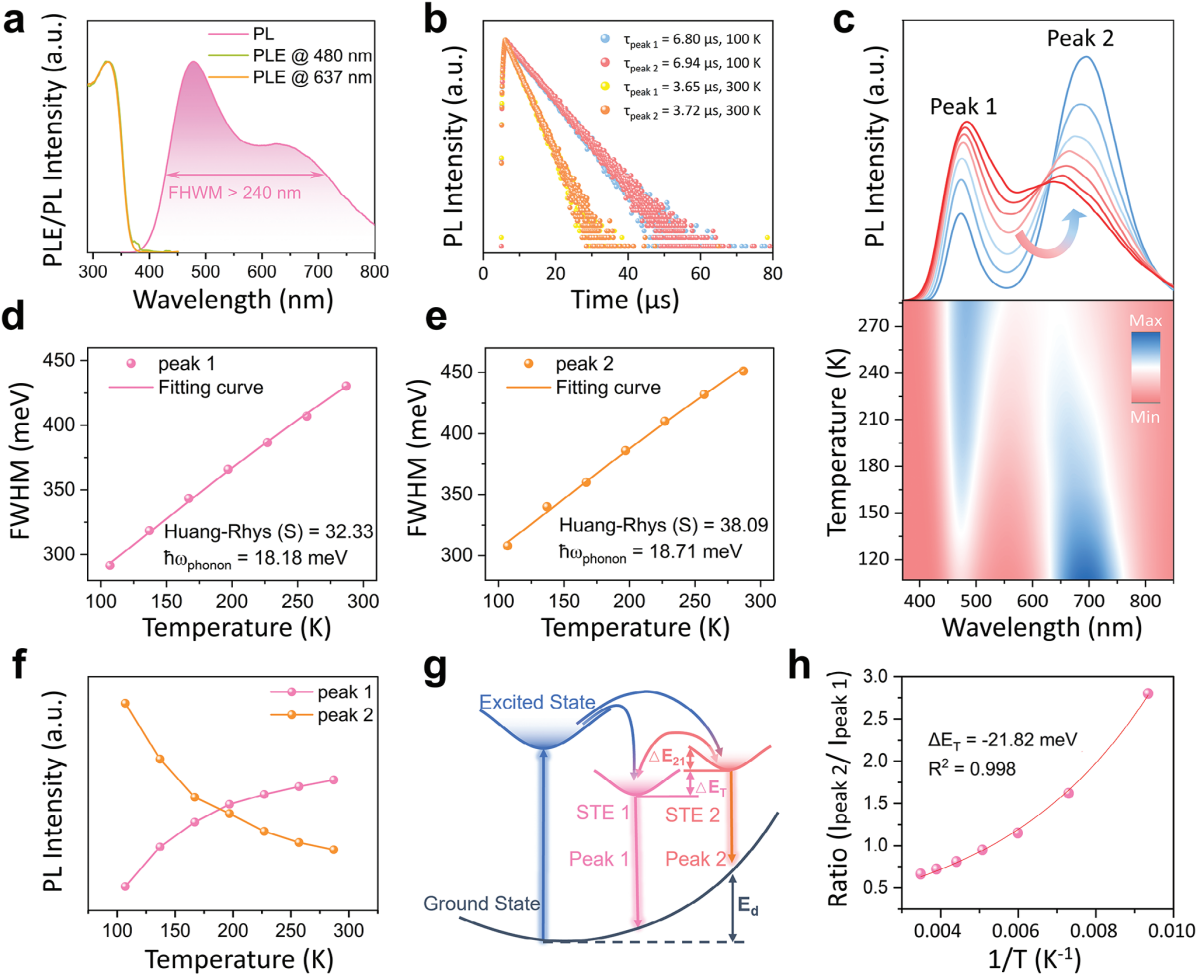
Photophysical characterization of the TPACI bulk crystals. a)PLE and PL emission spectra of the TPACI bulk crystals. b) Time‐resolved PL decay curves of TPACI bulk crystals under 325 nm excitation, monitored at 480 and 637 nm. c) PL spectra of TPACI bulk crystals measured at various temperatures ranging from 107 to 287 K. Temperature‐dependent evolution of FWHM of PL centered at 480 nm d) and 637 nm e). f) PL Emission intensity of peak 1 and peak 2 versus temperatures. g) Photophysical mechanism of TPACI. STE 1: emitting state 1; STE 2: emitting state 2; E_21_: the potential barrier going from STE 2 to STE 1; ΔE_T_: the potential energy difference between the STE 1 and STE 2 states; E_d_: lattice deformation energy. h) The ratio of peak 2/peak 1 as a function of temperature reciprocal (1/T).

The photoluminescence excitation (PLE) and photoluminescence (PL) emission spectra of the TPACI bulk crystals are shown in **Figure** **2**a. TPACI bulk crystals show distinct dual‐emission bands at 480 nm (peak 1) and 637 nm (peak 2) upon 325 nm excitation. The FWHM of the emission band can exceed 240 nm, covering from 435 to 681 nm, almost overlapping the visible region, which corresponds to its high‐quality white light emission. The PLE spectra of peak 1 and peak 2 were also measured, which shows a similar profile with an excitation peak centered at 327 nm. It can be observed a large Stokes shift of 153 nm for peak 1 and 310 nm for peak 2. This wide FWHM and large Stokes shift are similar to other low‐dimensional metal halides, which are considered to originate from STEs.^[^ ^25^ ^]^ It is worth noting that this large Stokes shift can effectively reduce self‐absorption to increase the light yield properties of the material. Because of these unique properties, such materials have great potential to be used in scintillator applications.^[^ ^13^ ^]^ The PLQY of TPACI bulk crystals is measured to be 94.27% (Figure S2, Supporting Information). Figure 2b presents the time‐resolved PL decay curves of TPACI bulk crystals probed at 480 and 637 nm, respectively, under excitation by a 325 nm laser at temperatures of 100 and 300 K. The single‐exponential equation can be used to fit the decay curves determined by exciton radiation character.^[^ ^26^, ^27^ ^]^ The TPACI bulk crystals exhibit a microseconds level decay lifetime at room temperature, which is further prolonged at temperatures down to 100 K.

To further investigate the emission mechanism of TPACI, Figure 2c presents the temperature‐dependent PL spectra of TPACI excited by a 335 nm laser. The broad two‐band emission can be detected in the entire temperature window, indicating no temperature‐dependent phase transition. The shift of the main PL peak is accompanied by a reduction of the FWHM at lower temperatures, which can be attributed to the reduction of electronic coupling with acoustic phonons.^[^ ^28^ ^]^ By fitting the temperature‐dependent FWHM using the following equation:

(2) $$FWHM\;=\;2.36\sqrt{S}\hbar \omega_{phonon}\sqrt{\coth\left(\hbar \omega_{phonon}/2k_{B}T\right)}$$

where ℏ is the reduced Planck constant, ω phonon frequency, T temperature, k_B_ Boltzmann constant, and S Huang–Rhys factor. The coth and T are positively correlated; as T decreases, coth decreases, leading to a lower FWHM.^[^ ^29^ ^]^ The S values of the peak 1 and peak 2 emission are calculated as 35.33 and 38.09, which is much larger than that of most of the conventional emission materials, such as CdSe, ZnSe, and CsPbBr_3_ (Figure 2d,e).^[^ ^30^, ^31^, ^32^ ^]^ The robust exciton‐phonon coupling observed in TPACI suggests that their crystal lattice is soft, making it highly conducive to triggering the emergence of self‐trapped excited states.^[^ ^33^, ^34^ ^]^ With increasing temperature, peak 1 shows a slight red shift and peak 2 shows an obvious blue shift. The red shift of peak 1 may be caused by the bandgap shrinkage due to the enhanced electron–phonon interactions. An important role in this blueshift is likely to be played by the variable lattice deformation and valence band shift with temperature.^[^ ^35^ ^]^ In general, STEs have a distinct thermally activated process, therefore the PL intensity of TPACI bulk crystals may be changed with the adjustment of the external temperature. With the increasing temperatures, the PL intensity of peak 1 keeps enhancing, whereas that of peak 2 shows an opposite trend (Figure 2f). Due to the cooling‐induced lattice contraction process, which enhances the metal‐metal interaction and reduces the probability of nonradiative decay, the emission intensity of peak 2 increases dramatically with decreasing temperature.^[^ ^36^ ^]^

STEs are common in halide crystals and organic molecular crystals.^[^ ^37^, ^38^ ^]^ In these solids, electron–phonon interactions are strong enough for excited electrons and holes to cause elastic distortions in the surrounding lattice. Thus, once electrons and holes are photogenerated, they quickly become self‐trapped, because the self‐trapped state is more stable than the one in which they would move, dragging the lattice distortion with them.^[^ ^39^ ^]^ An ideal model of the potential energy surface with two excited state energy minima is proposed for TPACI, as shown in Figure 2g, to describe the photophysical properties. The photophysical process for TPACI can be understood by considering the formation of STE states, the potential barrier between the two emitting states, and the thermal energy and exciton crossing from STE 2 to STE 1.^[^ ^40^ ^]^ A single TPA^+^ and [Cu_2_I_4_]^2−^ cluster binding together form the STE 1 state. Because of the short Cu–Cu distance (2.62 Å) in the TPACI, the adjacent STE 1 has a strong interaction, thus forming STE 2. Typically, lower temperatures tend to suppress nonradiative transitions and boost the luminescence intensity of copper‐based metal halides like (C_16_H_36_N)CuI_2_ and Cs_3_Cu_2_I_5_.^[^ ^17^, ^23^ ^]^ However, a strange behavior was observed in TPACI, where there was a notable increase in peak 1 intensity with increasing temperature. At low temperatures, some photoinduced carriers in the excited state relax more easily to STE 2 than to STE 1, producing a strong emission intensity of peak 2 and a relatively weak peak 1.^[^ ^27^ ^]^ With increasing temperature, the organic framework becomes softer and the excitons have a higher kinetic energy, which can promote excitons to overcome the potential barrier E_21_ and reach STE 1, so the intensity of peak 1 increases with temperature, while the corresponding peak 2 decays very quickly.^[^ ^23^ ^]^ The ratio of the emission intensity of peak 1 and peak 2 changes correspondingly at different temperatures, which is attributed to the varying distribution of excitons in the two emitting states.^[^ ^23^ ^]^ Since the formation of dual‐band emitting states is usually associated with structural distortions, a potential barrier between the two emitting states is predicted. The potential energy difference (ΔE_T_) between the STE 1 and STE 2 states can be obtained by fitting the ratio of STE 2 to STE 1 versus temperature using the following equation:^[^ ^41^ ^]^

(3) $$\frac{I_{STE\;2}}{I_{STE\;1}}=\;Ae^{-\Delta E_{T}/k_{B}T}$$

ΔE_T_ is calculated to be −21.82 meV, which indicates the minimum potential energy surface of STE 2 is higher than the one of STE 1. It is speculated that the energy of the ground state at STE 2 increases due to lattice deformation (E_d_), leading to the emergence of a distinct yellow‐emitting state. The photophysical mechanism of TPACI is different from the organic copper iodide reported previously.^[^ ^21^, ^40^, ^42^ ^]^

**Figure 3:**
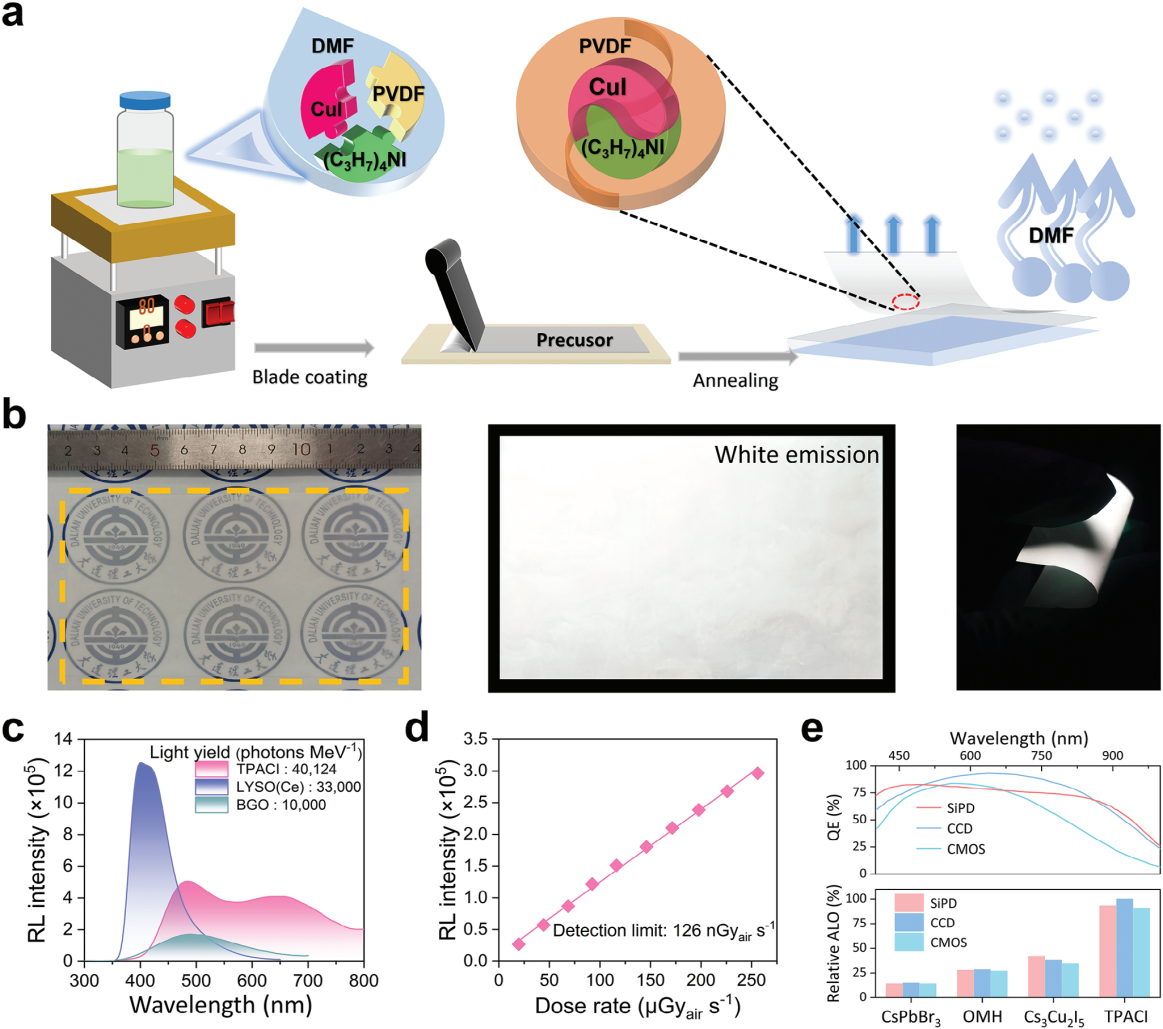
Scintillation performance of the TPACI membrane. a) Schematic depiction of the in situ synthesis of TPACI within the PVDF polymer. b) Photographs of the TPACI flexible membrane under ambient daylight and UV illumination. c) X‐ray‐induced RL spectra of the TPACI membrane, commercial reference LYSO: Ce and BGO scintillators (both thickness of 100 µm). d) RL intensity of the TPACI membrane as a linear function of the dose rates ranging from 20 to 258 µGy_air_ s^−1^. The detection limit was derived from the fitted line at an SNR of 3. e) Spectral response curves of CCD, CMOS, and SiPD photodetectors (top panel). The relative apparent light output of typical emerging metal halide scintillators in the case of RL peak being normalized (bottom panel). OMH: Organic manganese halides.

The TPACI membrane embedded within the polyvinylidene fluoride (PVDF) polymer was prepared by an in situ fabrication strategy to evaluate the scintillation performance. **Figure** **3**a illustrates a schematic of the in situ fabrication blade coating method. Tetrapropylammonium iodide (C_3_H_7_)_4_NI, cuprous (I) iodide (CuI), and PVDF were dissolved in an N, N‐dimethylformamide (DMF) to achieve the precursor solution. The precursor solution was then dropped onto a quartz substrate and smoothed with blade coating. A uniformly dispersed membrane was acquired after the volatilization of DMF and complete crystal crystallization. In this case, the addition of the PVDF polymer endows the solution with a high viscosity, allowing the crystal growth to be confined within a small and tight reaction chamber.^[^ ^43^ ^]^ The TPACI membrane was first performed with a series of material characterizations to demonstrate their crystallinity and elemental characterization in PVDF. As shown in Figure S3 (Supporting Information), the main X‐ray diffraction peaks in the TPACI membrane correspond to their bulk crystal counterparts with only some differences in diffraction intensity, proving the good crystallinity of the TPACI within the PVDF polymer. The FTIR results shown in Figure S1 (Supporting Information) reveal that the asymmetric C─H stretching and symmetric C─H stretching of the −CH_3_ group in the TPACI membrane are essentially consistent with their bulk crystal counterparts in the organic cation of TPA^+^, which further supports the existence of their identical coordination.^[^ ^22^ ^]^ Additionally, the characteristic bands at 841 and 1165 cm^−1^ are ascribed to the stretching frequencies of C–F in the TPACI membrane, corresponding to the vibrational bands at 869 and 1179 cm^−1^ in the pristine PVDF, respectively.^[^ ^44^ ^]^ Their C–F bending bands are located at 844 cm^−1^ in PVDF and 841 cm^−1^ in the TPACI NC membrane, respectively.^[^ ^45^ ^]^ XPS measurement was performed to identify elemental species of the TPACI membrane. Figure S4b (Supporting Information) shows the high‐resolution XPS results of C 1*s*, F 1*s*, Cu 2*p*, and I 3*d* in the TPACI membrane. Compared with that of the pristine PVDF sample (Figure S4c, Supporting Information), the C─C and C─H bond intensity of the TPACI membrane is significantly enhanced, which is ascribed to the large amount of propyl contained in the TPA^+^ cations. The F 1*s* peak exhibits a 0.2 eV shift from 686.5 eV for the TPACI membrane to 686.3 eV for the pristine PVDF membrane. This is due to the partial defluorination of PVDF, suggesting the possible interactions between TPA^+^ and ‐CF_2_‐. Similar XPS results of the C─C bond enhancement and F 1*s* peak shift have been reported in previous related publications.^[^ ^43^ ^]^ The Cu 2*p* and I 3*d* peaks of the TPACI membrane are in good agreement with their bulk crystal counterparts (Figure S4a, Supporting Information).

A photograph of the as‐prepared TPACI membrane is shown on the left of Figure 3b, where the university's insignia can be clearly observed, revealing the intuitive transparency due to weak optical scattering. Under UV excitation, the TPACI membrane shows a bright white emission (Figure 3b middle). Taking advantage of the characteristics of the PVDF polymer, the composite membrane showcases remarkable flexibility (Figure 3b right). It can be bent freely without breaking and effortlessly returns to its initial shape once the external force is removed. To evaluate the X‐ray performance of the TPACI membrane, two commercial scintillators, LYSO: Ce and BGO, were used as standard references, all with thicknesses of 100 µm and uniform geometries and dimensions. The light yields of the LYSO: Ce and BGO scintillators are known to be 33000 and 10000 photons per MeV, respectively. By integrating their X‐ray‐induced RL spectra and comparing the results, the light yield of the TPACI membrane can be calculated to be 40124 photons MeV^−1^ (Figure 3c). The RL intensity of the TPACI membrane as a function of the incident X‐ray dose rate is shown in Figure 3d and Figure S5 (Supporting Information). The fitted linear relationship manifests that the contrast of the X‐ray images is critically dependent on the X‐ray dose. The minimum detectable dose rate for X‐ray detection is demonstrated to be 126 nGy_air_ s^−1^ at SNR = 3, which is much lower than that required for conventional computed tomography (5.5 µGy_air_ s^−1^).^[^ ^46^ ^]^

To more visually present the advantages of broad‐emission TPACI scintillator, the spectral response compatibility of several emerging metal halides with different typical photodetectors (CCD, CMOS, and SiPD) was compared in the context of normalized RL intensity of these scintillators (Figure 3e). The RL spectra of these several emerging scintillators are available in Figure S6 (Supporting Information). As shown in Figure 3e (bottom panel), the relative apparent light output (ALO) was used as a parameter for comparison, as described in the introduction.^[^ ^17^, ^47^, ^48^, ^49^ ^]^ It can be seen that the broad‐emission spectrum of TPACI exhibits a more universal compatibility with the spectral response of different photodetectors. This demonstrates that the broad‐emission TPACI scintillator is expected to provide high output signals for photodetectors due to its high scintillation light collection efficiency in the integration of detectors.

**Figure 4:**
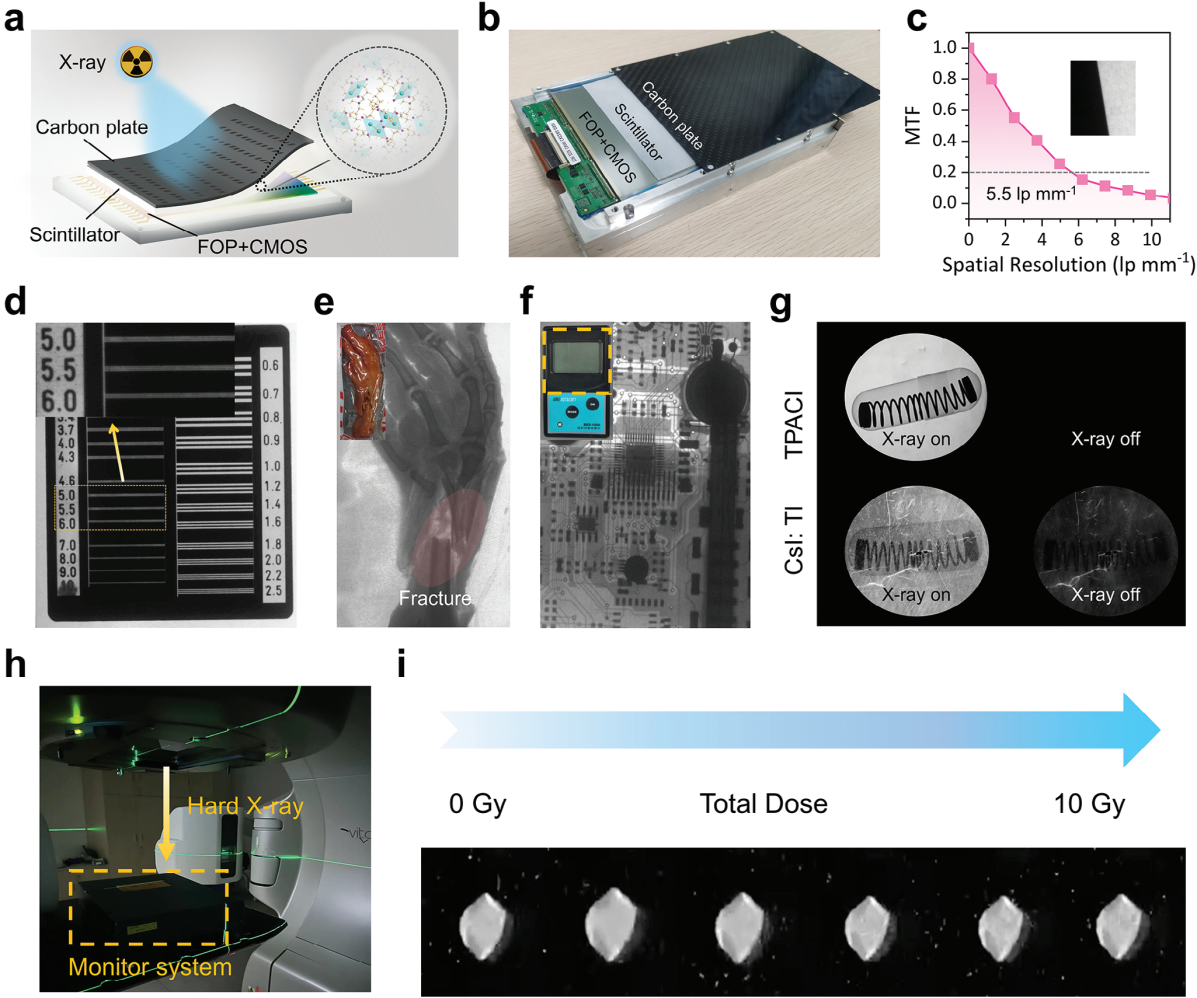
TPACI scintillator‐based X‐ray imager. a) A schematic device structure and b) the practical photo of the TPACI scintillator‐based X‐ray imager. c) MTF curves of the TPACI scintillator‐based X‐ray imager. The inset shows the X‐ray edge image used for MTF calculation. d) X‐ray image of the X‐ray standard pattern chart. The inset shows the magnification view within the yellow box. Bright‐field (inset) and X‐ray images of a chicken claw e) and a dosimeter f). g) Photographs of the TPACI membrane and CsI: Tl scintillation light with X‐rays on (left) and off (right) at consecutive 2 s exposures. h) A Photograph of irradiation stability tests on a medical radiotherapy accelerator. i) TPACI scintillation light output images with increasing dose rate.

The TPACI scintillator membrane is further assembled into an X‐ray imager for X‐ray imaging demonstrations. **Figure** **4**a,b depict the schematic device architecture and the practical photo of the TPACI scintillator‐based X‐ray imager, consisting of a carbon sheet, a TPACI scintillator membrane, and an arrayed CMOS sensor module covered by a fiber optic plate (FOP) layer. Benefiting from the robust RL emission due to the broad spectrum and the high homogeneity of TPACI scintillator membrane, the TPACI scintillator‐based X‐ray imager exhibits a high spatial resolution of 5.5 lp mm^−1^ at MTF = 0.2 (Figure 4c), which is higher than that of Cs_3_TbCl_6_ (3.3 lp mm^−1^ at MTF = 0.2) and Rb_3_TbCl_6_ scintillator‐based detectors (3.9 lp mm^−1^ at MTF = 0.2) in the similar experimental configuration from the previous publication.^[^ ^50^ ^]^ As shown in Figure 4d, a spatial resolution greater than 6.0 lp mm^−1^ in the standard line chart was clearly distinguished. To further validate the X‐ray imaging resolution of the TPACI membrane, some objects were used as imaged samples. Figure 4e,f show the bright‐field and X‐ray images of a chicken claw and a dosimeter. The fracture of chicken claw bones and the details of the internal circuitry in the dosimeter were clearly resolved without blurring. In addition, Figure 4g shows the RL photographs of the TPACI membrane and CsI: Tl with X‐ray on and off during a continuous 2 s exposure time. A clear afterglow light was still observed in CsI: Tl when the luminescence of the TPACI membrane completely decayed to dark background images. Actually, characteristic afterglows were usually found in dopant‐activated scintillators, such as a few hours of afterglow in LYSO: Ce.^[^ ^51^ ^]^ It is worth noting that the frames per second (FPS) for existing commercial DDR flat‐panel detector products typically range from 1 to 100. The low afterglow levels of the TPACI membrane make it particularly attractive for high‐FPS DDR applications. To verify the stability of the TPACI scintillator under strong irradiation, it was placed on a medical radiotherapy accelerator subjected to hard X‐ray irradiation, and the evolution of its light output was monitored (Figure 4h; Figure S7, Supporting Information). Figure 4i shows that the RL signal response of a TPACI scintillator demonstrates no obvious degradation under hard X‐ray (energy of 6 MeV) irradiation with a cumulative dose of 10 Gy, implying it can maintain reliable and consistent scintillation performance under prolonged radiation exposure.

## 3 Conclusion

In summary, we investigated TPACI organic copper halide with efficient dual‐band white light emission and synthesized membrane using an in situ solution fabrication strategy for applications in X‐ray imaging. This scintillator has been demonstrated to offer a multitude of advantages, including flexibility, high light yield, non‐toxicity, a low detection limit, and a low afterglow level. Most importantly, the broad‐emission feature demonstrates universal compatibility with the spectral response of commercial photodetectors. In addition, the TPACI scintillator demonstrates excellent irradiation stability during exposure to 6 MeV hard X‐rays with a cumulative dose of 10 Gy from the medical radiotherapy accelerator. As a proof of concept, a prototype of a TPACI scintillator‐based X‐ray imager was constructed to inspect the internal structure of biological and electronic device. It demonstrates an outstanding scintillation performance with a spatial resolution of 5.5 lp mm^−1^ at MTF = 0.2. This work contributes to the progress of non‐toxic scintillators in high‐resolution X‐ray imaging applications.

## Conflict of Interest

The authors declare no conflict of interest.

## Supporting information

Supporting Information

## Data Availability

The data that support the findings of this study are available from the corresponding author upon reasonable request.
