# Supplementary material for: Ultra‐Broad Emission Copper Halide Scintillator‐Based X‐Ray Imager
Source: Adv Sci (Weinh). 2024 Nov 26;12(4):2405995. doi: 10.1002/advs.202405995 (PMC11789579; doi:10.1002/advs.202405995)
Supplement: Supplementary file 1 — Supporting Information [file ADVS-12-2405995-s001.docx]

Supporting Information

**Ultra-Broad Emission Copper Halide Scintillator-Based X-Ray Imager**

*Haocheng Lv,^†^ Wenvi Shao,^†^ Haifeng Chen, Guoyang Zhu, Yao Wang, Zhenzhong Zhang,^*^ Hongwei Liang^*^*

**Experimental Section**

**Synthesis of TPACI Bulk Crystals.** The bulk crystals of TPACI were prepared via a solution slow evaporation method. First, 8 mmol of CuI and 8mmol of (C_3_H_7_)_4_NI were dissolved in 6 mL of N, N-dimethylformamide (DMF) under continuous stirring at 100 °C, forming a clear precursor solution. The as-prepared solution was slowly evaporated the solvent at room temperature to harvest colorless and Transparent crystals after 3 to 5 days.

**In Situ Synthesis of the TPACI Membrane**. A precursor solution was prepared by direct mixing 5 mmol of (C_3_H_7_)_4_NI, 5mmol of CuI, and 1.5 g of polyvinylidene fluoride (PVDF) powder in 5 mL of a DMF solvent and was magnetically stirred at 100 °C until it was completely dissolved into a transparent solution. The above-mentioned precursors were dropped onto a glass substrate (in advance, the glass substrate was sonicated with acetone, ethanol, and deionized water for 30 min) and smoothed out with a blade-coating method. Then, the pre-formed membrane was placed on a hotplate at 120 °C for an annealing treatment to promote the crystallization of the TPACI within the polymer PVDF. After solvent evaporation and full crystallization of TPACI, this membrane showed a uniform and bright white light emission under UV lamp excitation, at which point it could be uncovered from the glass substrate.

**Characterizations.** Single-crystal X-ray diffraction (SCXRD) was measured using a Rigaku XtalAB PRO MM007DW X-ray diffractometer. Powder XRD patterns were characterized using a D/max-Ultima+ X-ray diffractometer. X-ray photoelectron spectroscopy (XPS) was carried out using Thermo Fisher ESCALAB Xi+. Fourier-transform infrared spectroscopy (FTIR) was carried out with a Thermo Scientific Nicolet iS20. PL and PL excitation (PLE) spectra were measured using a spectrometer (Edinburgh, FLS1000). Time-resolved PL (TRPL) was conducted using a spectrometer (Edinburgh FLS980) equipped with a laser.

**X-ray Scintillation Performance**

The radioluminescence (RL) spectra were recorded employing a spectrometer (Horiba Fluromax-4) equipped with an X-ray tube (Tungsten target, Moxtek, ULTRA-LITE MAGNUM). The detection slit was set at 5 nm, and the X-ray outlet was set to 1 cm away from the sample. Dose rate-dependent RL curves were obtained by changing the tube current from 1 to 10 μA at a fixed tube voltage (50 kV). The slope was derived from the linear relationship between the RL intensity versus the X-ray dose rate. The detection limit was calculated using the 3σ/slope method, where the σ is the average noise intensity. The noise data was recorded in the background without samples, and then statistically analyzed and fitted by a Gaussian function, where the full width at half maximum was used as the average value of the noise.

**X-ray imaging**

The TPACI scintillator membrane was air-coupled directly to a small CMOS detector (Shad-o-Box 3K HS, Teledyne Dalsa) and pressed through a carbon plate and sponge, assembling a X-ray imager. The effective area of the detector is 11.4 × 6.4 mm with a pixel size of 49.5 µm. The MTF curve was measured by the slanted-edge method. The MTF calculation was performed by the plug-in SE_MTF_2xNyquis installed in the Image J software.

**Computational Methods.** We utilized the Vienna Ab Initio Package (VASP) for conducting density functional theory (DFT) calculations within the generalized gradient approximation (GGA) using the PBE formulation. The ionic cores were described using projected augmented wave (PAW) potentials, and valence electrons were taken into account using a plane wave basis set with a kinetic energy cutoff of 400 eV. Partial occupancies of the Kohn−Sham orbitals were incorporated through the Gaussian smearing method with a width of 0.05 eV. Self-consistency in electronic energy was achieved when the energy change was below 10−5 eV. The geometry optimization was considered converged when the force change was less than 0.02 eV/Å. Grimme’s DFT-D3 methodology was employed to characterize dispersion interactions.

Table S1 Single crystal X-ray diffraction data of TPA2Cu2I4

| Compound | TPA2Cu2I4 |
| --- | --- |
| Formula sum | C24 H56 Cu2 I4 N2 |
| Formula weight | 1007.38 g/mol |
| Crystal system | monoclinic |
| Space-group | P 1 21/n 1 (14) |
| Cell parameters | a=8.9397(2) Å  b=12.4726(3) Å  c=16.0447(5) Å  β=92.242(2)° |
| Cell ratio | a/b=0.7167  b/c=0.7774  c/a=1.7948 |
| Cell volume | 1787.64(8) Å^3^ |
| Z | 2 |
| Calc. density | 1.8714 g/cm^3^ |
| RAll | 0.0262 |
| Pearson code | mP176 |
| Formula type | NOP2Q12R28 |
| Wyckoff sequence | e44 |


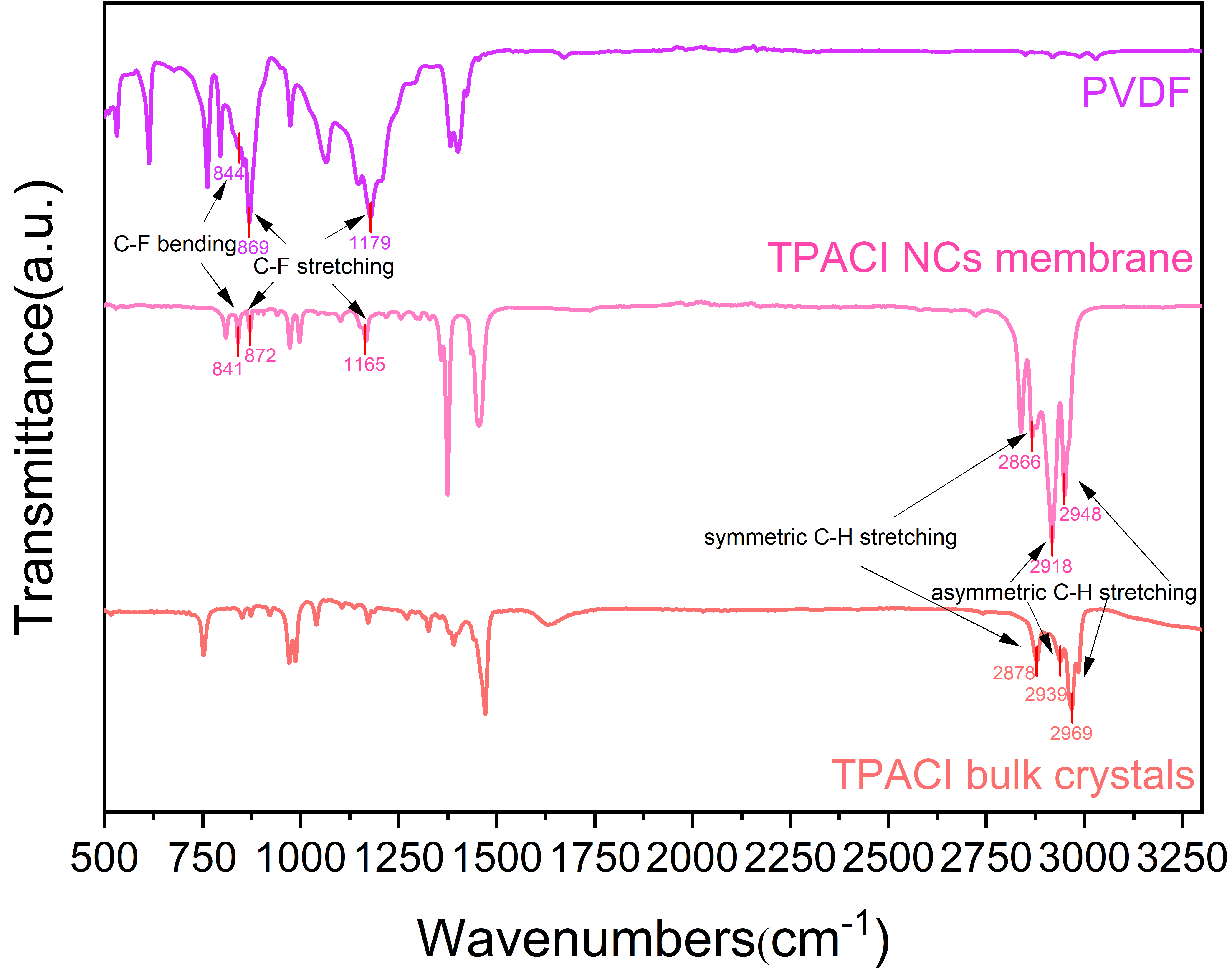


Figure S1. Fourier transform infrared spectroscopy of pure PVDF, TPACI membrane and TPACI bulk crystals.


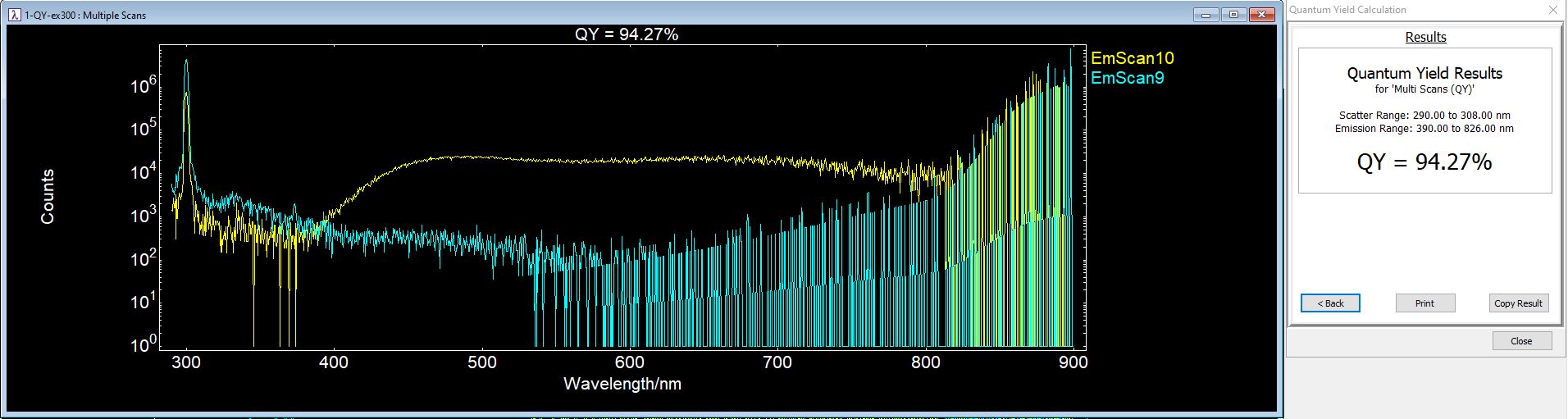


Figure S2. PLQY of TPACI bulk crystals under 300 nm excitation.


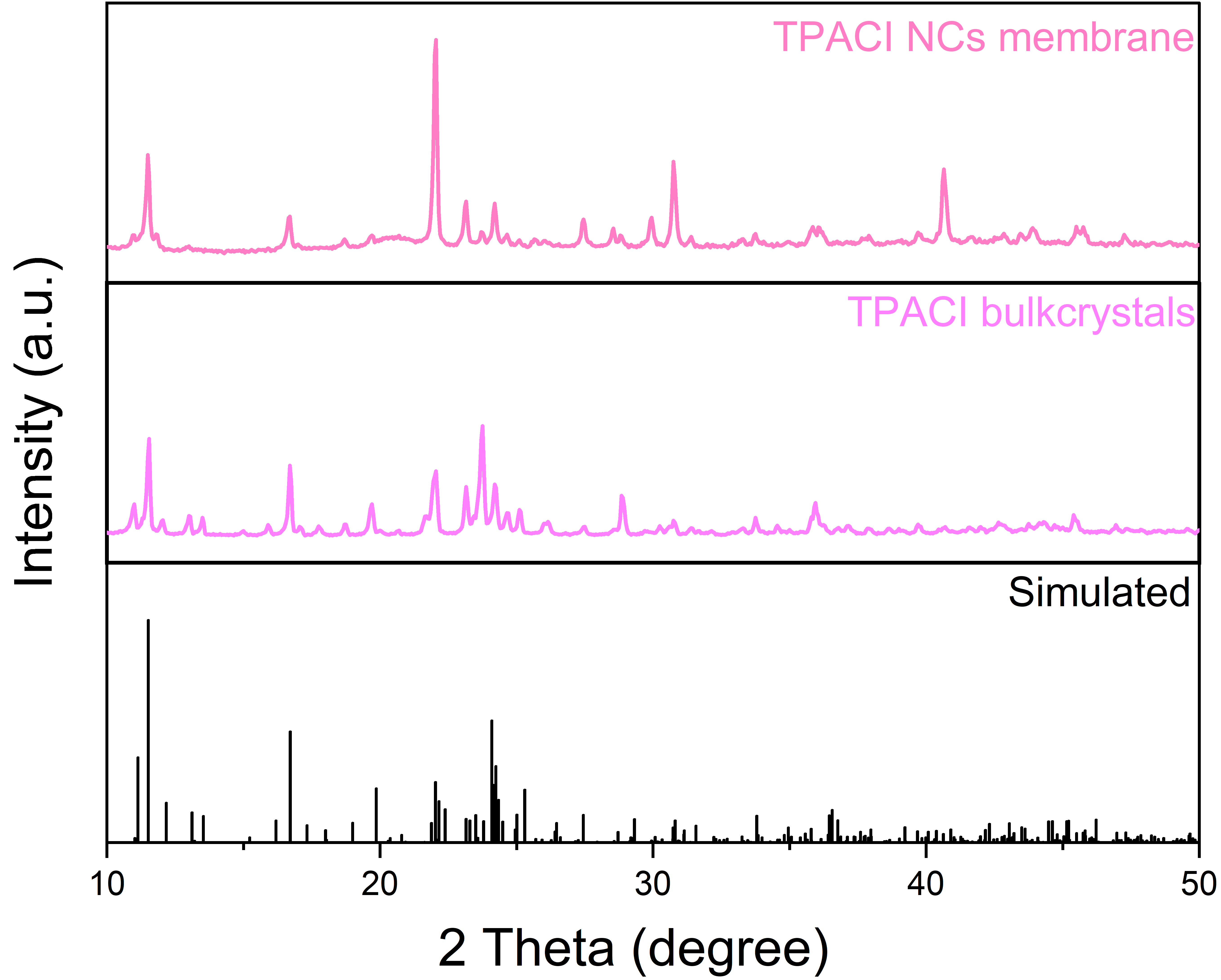


Figure S3 PXRD patterns of the TPACI membrane, bulk crystals, and the corresponding simulated results from the single-crystal structure.


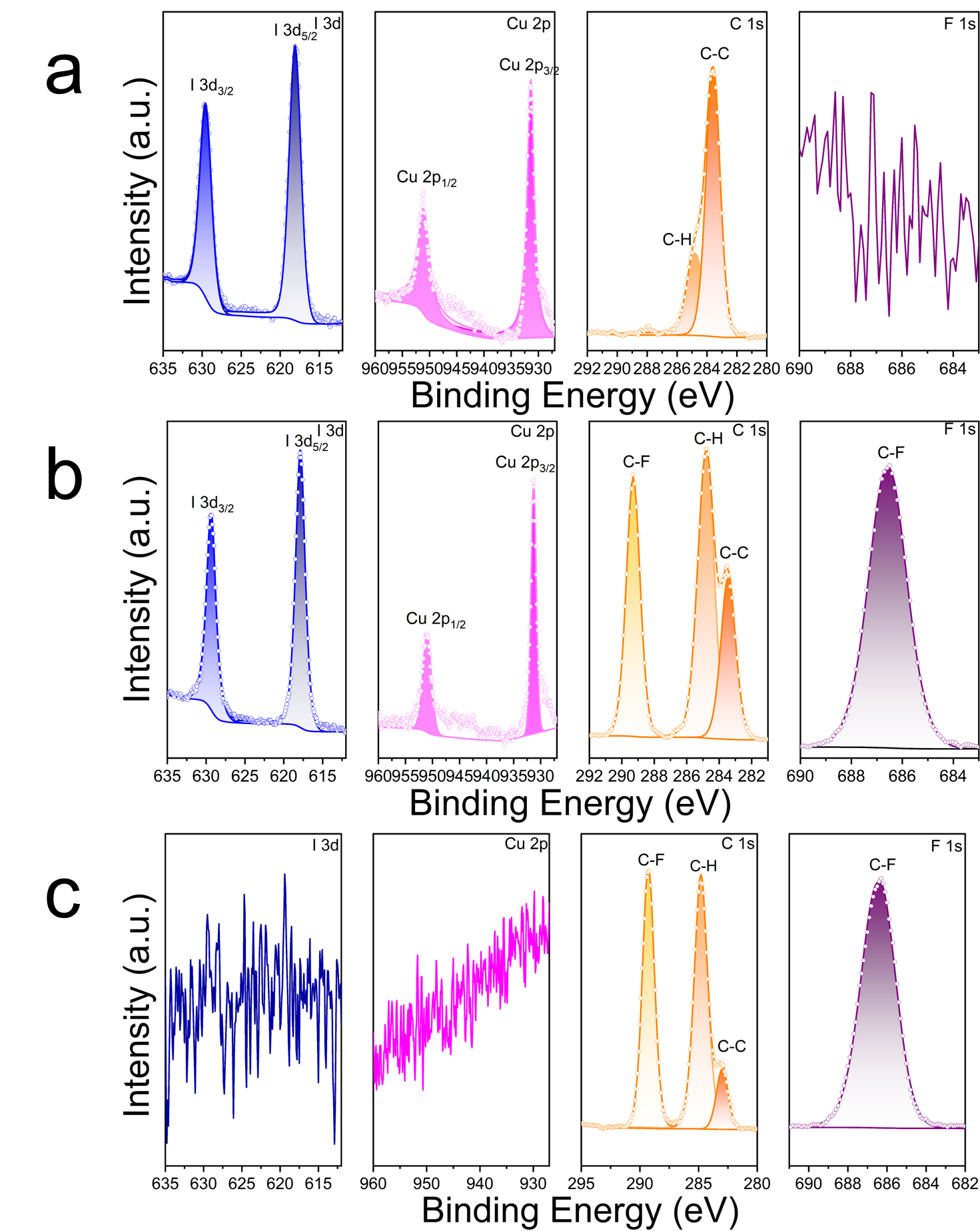


Figure S4 XPS of C 1s, F 1s, Cu 2p, and I 3d in TPACI bulk crystals (a), membrane (b) and pristine PVDF (c).


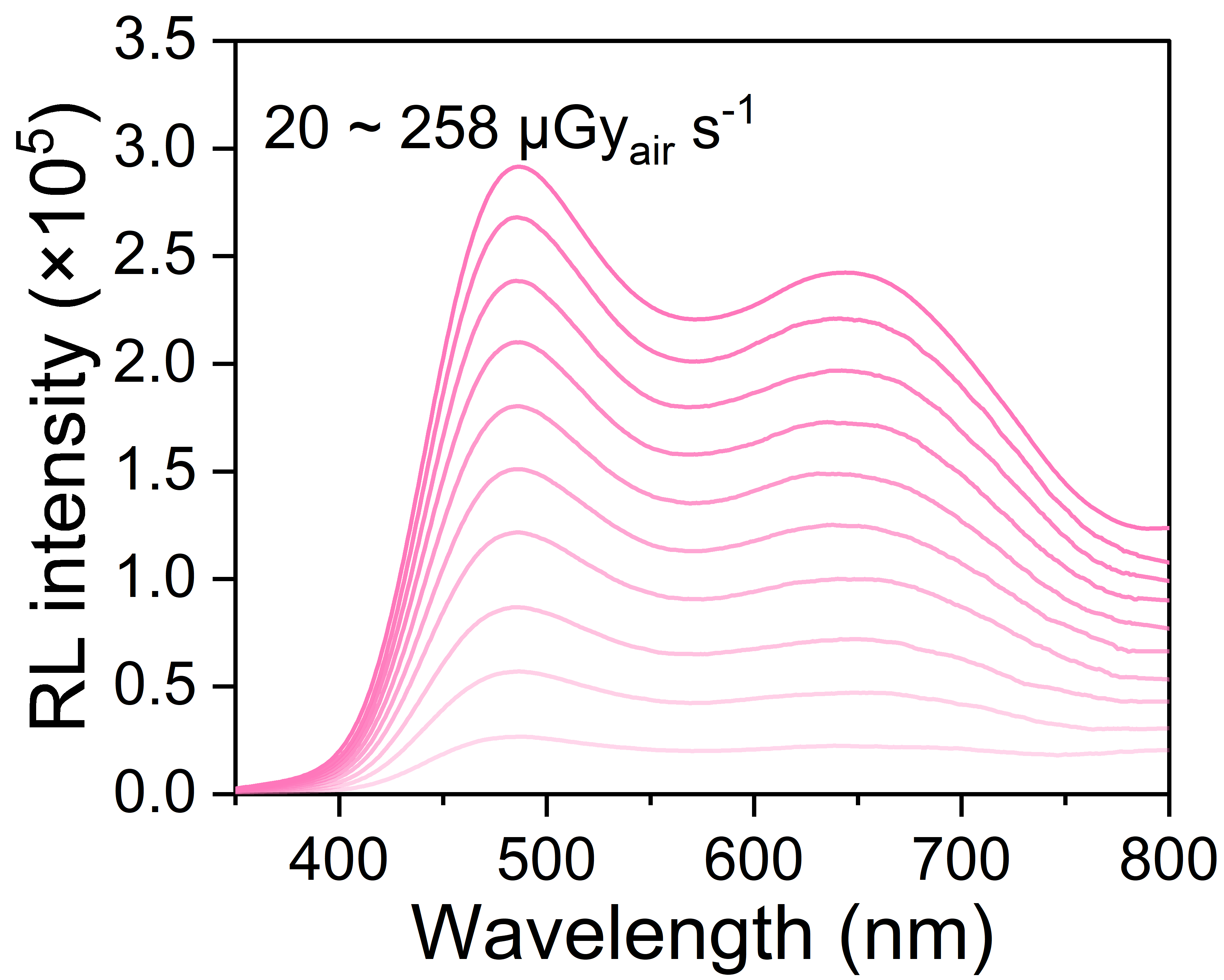


Figure S5 Dose rate-dependent RL spectra of the TPACI membrane at the dose rates ranging from 20 to 258 μGy_air_ s^-1^.


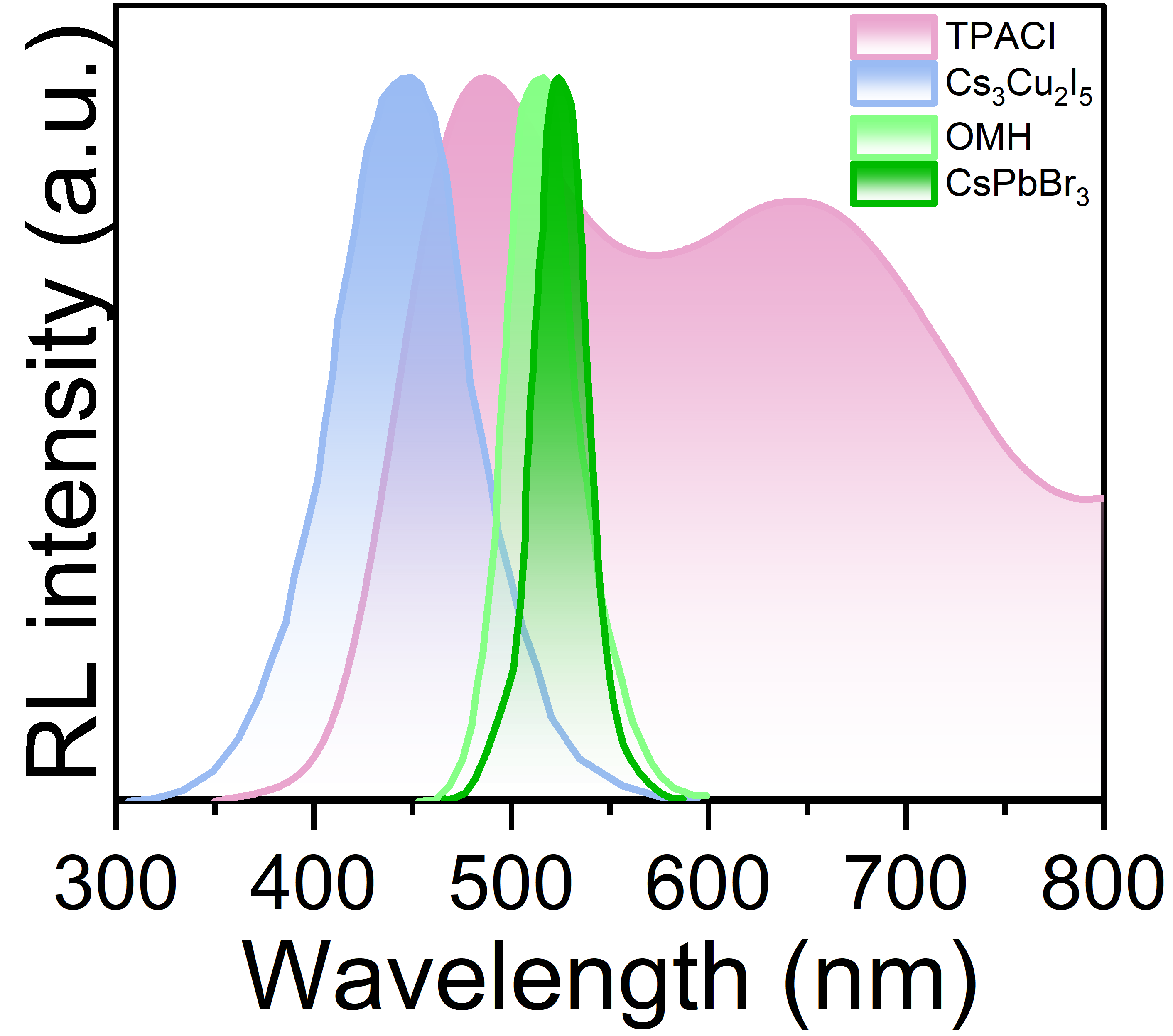


Figure S6 RL spectra of CsPbBr_3_, OMH and Cs_3_Cu_2_I_5_.


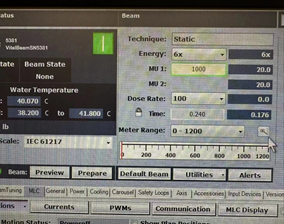


Figure S7 Setup parameters for irradiation stability testing on a medical radiotherapy accelerator.
